# Redox Signaling in Degenerative Diseases: From Molecular Mechanisms to Health Implications

**DOI:** 10.1155/2014/245761

**Published:** 2014-06-25

**Authors:** Cristina Angeloni, Tullia Maraldi, David Vauzour

**Affiliations:** ^1^Department for Life Quality Studies, Alma Mater Studiorum University of Bologna, 47921 Rimini, Italy; ^2^Department of Surgical, Medical, Dental and Morphological Sciences with Interest in Transplant, Oncology and Regenerative Medicine, University of Modena and Reggio Emilia, 41124 Modena, Italy; ^3^Norwich Medical School, Faculty of Medicine and Health Sciences, University of East Anglia, Norwich NR4 7TJ, UK

Maintenance of normal intracellular redox status plays an important role in regulating many physiological processes. The cellular oxidation and reduction environment is influenced by the production and removal of reactive oxygen species (ROS). Unbalanced levels of ROS are a common characteristic of many acute and chronic degenerative diseases such as cancer, cardiovascular diseases, type II diabetes, acute liver and renal failure, and neurodegenerative disorders including Parkinson's and Alzheimer's diseases and strokes. On the other hand, in the last years it has been shown that not only are ROS detrimental to cells but at physiological level they regulate a myriad of cellular processes including transcription regulation and cell signaling. Several reports support the hypothesis that cellular ROS levels could function as “second messengers.” The second messenger properties of ROS are believed to activate signaling pathways by regulating kinases, phosphatases, transcription factors, or ion channels to coordinate the final response of the cell. Understanding the crosstalk between signaling, ROS, and cell homeostasis is fundamental for understanding redox biology and disease pathogenesis.

This special issue provides a selection of original articles as well as reviews focused on the role of redox signaling in different cell systems and pathological conditions.

A role for intracellular ROS production has been recently implicated in the pathogenesis and progression of a wide variety of neoplasias. ROS sources, such as NAD(P)H oxidase (Nox) complexes, are frequently activated in acute myeloid leukemia blasts and strongly contribute to proliferation, survival, and drug resistance of these cells. M. Guida et al. investigated the role of nuclear Nox-derived ROS in myelodysplastic syndromes (MDS). They reported, in human MDS samples, that Nox4 isoform is localized into the nucleus. Moreover, they demonstrated Nox4 presence in speckle domains proposing that Nox4 could be involved in regulating DNA-mRNA processing machinery by ROS production in specific nuclear area and that Nox4 interacts with Akt and ERK signaling, suggesting a role of nuclear signaling disregulation in MDS progression.

J. K. Kwee explored the paradoxical double effect of antioxidants in solid cancer cells. Antioxidants may hamper the efficacy of chemotherapy by scavenging reactive oxygen species and, on the other hand, alleviate unwanted chemotherapy-induced toxicity, thus allowing for increased chemotherapy doses. From this point of view, the modulation of intracellular antioxidant concentration is a double-edged sword, with both sides exploited for potential therapeutic benefits.

Caveolae/lipid rafts are membrane-rich cholesterol domains endowed with several functions in signal transduction and caveolin-1 (Cav-1) has been reported to be implicated in regulating multiple cancer-associated processes, ranging from tumor growth to multidrug resistance and angiogenesis. As vascular endothelial growth factor receptor-2 (VEGFR-2) and Cav-1 frequently colocalize, C. Caliceti et al. investigated the presence of VEGFR-2 in caveolae/lipid rafts in a leukemia cell line. Results demonstrated that caveolae/lipid rafts act as platforms for negative modulation of VEGF redox signal transduction cascades leading to glucose uptake and cell proliferation, suggesting therefore novel potential antileukemia targets.

Persistent inflammatory and oxidative stresses are hallmarks of most chronic degenerative diseases such as cardiovascular diseases and CNS pathologies (Alzheimer's, ALS). T. B Kuhn observed that both TNF*α* and Il-1*β* impaired morphology and motility of growth cones in spinal cord neuron cultures. Interestingly, inhibiting NADPH oxidase activity rescued loss of neuronal motility and morphology, suggesting that NADPH oxidase serves as a pivotal source of oxidative stress in neurons and, together with a disruption of actin filament reorganization, contributes to the progressive degeneration of neuronal morphology in the diseased or aging CNS.

Mild cognitive impairment (MCI) is regarded as a prodromal phase of late onset Alzheimer's disease (LOAD). It has been proposed that oxidative stress might be implicated in the pathogenesis of LOAD. C. Cervellati et al. investigated whether a redox imbalance, measured as serum level of hydroperoxides and/or serum antioxidant capacity, might be predictive of the clinical progression of MCI to LOAD. Their results suggested that oxidative stress might be precociously involved in LOAD pathogenesis but the two markers are not able to predict the progression from MCI to LOAD.

The review of C. Angeloni et al. focused on the new emerging role of advanced glycation end products (AGEs), induced by high glucose levels or methylglyoxal (MG), in the pathogenesis of Alzheimer's disease (AD). AGEs extensively cross-link proteins in A*β* deposits and neurofilaments exacerbating AD pathological hallmarks. Moreover, AGEs and MG are neurotoxic mediators of oxidative stress in the progression of AD and are capable of activating many redox signaling pathways such as ERK1/2, JNK, and p38 MAPK leading to apoptosis and cellular dysfunction.

Peroxisomes provide glial cells with protective functions against the harmful effects of H_2_O_2_ on neurons and peroxisome impairment results in nervous lesions. The study of L. D. C. Mannelli et al. highlighted that the PPAR-*γ* block in astrocytes is strictly related to reduced catalase functionality and expression with a general decrease in antioxidant defenses of the cell. The relevance of the damage induced by PPAR-*γ* impairment suggests that hypofunctionality of this receptor in glial cells could be present in neurodegenerative diseases and participate in pathological mechanisms through peroxisomal damage.

The review of C. Caliceti et al. describes how ROS regulate Notch and Wnt pathways in the cardiovascular system. Scientific evidences show a sequential and direct link between Notch and Wnt signaling pathways in tuning endothelial cells (ECs), cardiomyocytes functions, and vascular morphogenesis. Understanding the molecular mechanism regulated by ROS could lead to the development of new therapeutic approaches for cardiovascular diseases.

The so-called “glucose spikes,” mainly occurring after meals, confer a high cardiovascular risk attributed to acute increases of oxidative stress. The study of I. Russo et al. mimicked “the glucose spikes” in vitro incubating vascular smooth muscle cells (VSMC) with high glucose and demonstrating that high glucose, via oxidative stress, can reduce the cardiovascular protection conferred by the NO/cGMP pathway via phosphorylation of the cytoskeleton protein VASP in VSMC.

The cross-sectional population-based study of C. Cervellati et al. was conducted to investigate the role of oxidative stress in the derangement of bone homeostasis, a hallmark of postmenopausal osteoporosis (PO). They showed an association between increased hydroperoxides serum levels and reduced bone density in postmenopausal women, suggesting that oxidative stress might play a role in the development of PO by enhancing bone resorption rate.

Redox imbalance of luminal pyridine nucleotides in the endoplasmic reticulum (ER) together with oxidative stress results in the activation of autophagy. O. Kapuy and G. Bánhegyi demonstrated that the depletion of ER NADPH in HepG2 cell line, either by the pharmacological agent metyrapone or by silencing the key proteins of luminal NADPH generation, switched on an autophagic mechanism with the incomplete inactivation of the mTOR pathway.

Fibrogenesis is widely considered as the result of a dysregulated wound healing response. In particular, failure of the wave of myofibroblast apoptosis during wound healing combined with an autocrine feed-forward loop of TGF-*β* production leads to the development and persistence of large numbers of myofibroblasts, a hallmark of fibrotic disorders. The review of N. Sampson et al. summarizes recent in vitro and in vivo data demonstrating that TGF-*β*-induced myofibroblast differentiation is driven by a prooxidant shift in redox homeostasis.

Collectively, the papers of this special issue provide a better understanding of the role of redox signaling in the pathophysiology of different degenerative diseases and we hope that these contributions could encourage further studies in this important field.

*Cristina Angeloni* *Tullia Maraldi* *David Vauzour*

